# Supplementary material for: Modeling glioblastoma heterogeneity as a dynamic network of cell states
Source: Mol Syst Biol. 2021 Sep 16;17(9):e10105. doi: 10.15252/msb.202010105 (PMC8444284; doi:10.15252/msb.202010105)
Supplement: Supplementary file 5 — Source Data for Figure 3 [file MSB-17-e10105-s001.zip › Figure3A_sourcedata/GSEA_3065/hallmarks_state5.GseaPreranked.1623416302988/HALLMARK_IL2_STAT5_SIGNALING.html]

Details for gene set HALLMARK\_IL2\_STAT5\_SIGNALING[GSEA]

|  || Dataset | state5 |
| Phenotype | NoPhenotypeAvailable |
| Upregulated in class | na\_pos |
| GeneSet | HALLMARK\_IL2\_STAT5\_SIGNALING |
| Enrichment Score (ES) | 0.3050051 |
| Normalized Enrichment Score (NES) | 1.2339212 |
| Nominal p-value | 0.10552764 |
| FDR q-value | 0.17614108 |
| FWER p-Value | 0.702 |
Table: GSEA Results Summary

  

Fig 1: Enrichment plot: HALLMARK\_IL2\_STAT5\_SIGNALING      
 Profile of the Running ES Score & Positions of GeneSet Members on the Rank Ordered List

  

| PROBE | GENE SYMBOL | GENE\_TITLE | RANK IN GENE LIST | RANK METRIC SCORE | RUNNING ES | CORE ENRICHMENT || 1 | WLS |  |  | 116 | 0.509 | 0.0164 | Yes |
| 2 | ITGAV |  |  | 118 | 0.508 | 0.0457 | Yes |
| 3 | CD81 |  |  | 159 | 0.475 | 0.0687 | Yes |
| 4 | ALCAM |  |  | 225 | 0.422 | 0.0858 | Yes |
| 5 | EMP1 |  |  | 258 | 0.404 | 0.1055 | Yes |
| 6 | SYT11 |  |  | 265 | 0.400 | 0.1280 | Yes |
| 7 | CD44 |  |  | 378 | 0.349 | 0.1356 | Yes |
| 8 | COL6A1 |  |  | 536 | 0.301 | 0.1353 | Yes |
| 9 | ITGA6 |  |  | 552 | 0.297 | 0.1508 | Yes |
| 10 | IGF1R |  |  | 557 | 0.295 | 0.1675 | Yes |
| 11 | IGF2R |  |  | 705 | 0.260 | 0.1660 | Yes |
| 12 | SPP1 |  |  | 724 | 0.256 | 0.1788 | Yes |
| 13 | LRIG1 |  |  | 754 | 0.250 | 0.1900 | Yes |
| 14 | PHTF2 |  |  | 790 | 0.243 | 0.2001 | Yes |
| 15 | P4HA1 |  |  | 792 | 0.242 | 0.2140 | Yes |
| 16 | PLEC |  |  | 824 | 0.237 | 0.2242 | Yes |
| 17 | NT5E |  |  | 834 | 0.235 | 0.2368 | Yes |
| 18 | PRNP |  |  | 851 | 0.232 | 0.2484 | Yes |
| 19 | IFNGR1 |  |  | 983 | 0.213 | 0.2460 | Yes |
| 20 | BMPR2 |  |  | 1013 | 0.209 | 0.2548 | Yes |
| 21 | TWSG1 |  |  | 1016 | 0.208 | 0.2666 | Yes |
| 22 | SNX14 |  |  | 1048 | 0.204 | 0.2750 | Yes |
| 23 | LCLAT1 |  |  | 1116 | 0.196 | 0.2788 | Yes |
| 24 | ADAM19 |  |  | 1173 | 0.188 | 0.2833 | Yes |
| 25 | SLC2A3 |  |  | 1209 | 0.183 | 0.2900 | Yes |
| 26 | IFITM3 |  |  | 1332 | 0.168 | 0.2860 | Yes |
| 27 | COCH |  |  | 1343 | 0.167 | 0.2945 | Yes |
| 28 | POU2F1 |  |  | 1467 | 0.156 | 0.2897 | Yes |
| 29 | PTRH2 |  |  | 1498 | 0.153 | 0.2951 | Yes |
| 30 | PTCH1 |  |  | 1526 | 0.150 | 0.3007 | Yes |
| 31 | XBP1 |  |  | 1564 | 0.145 | 0.3050 | Yes |
| 32 | CKAP4 |  |  | 1636 | 0.139 | 0.3050 | Yes |
| 33 | FAM126B |  |  | 1851 | 0.123 | 0.2880 | No |
| 34 | PLPP1 |  |  | 2048 | 0.108 | 0.2722 | No |
| 35 | SYNGR2 |  |  | 2623 | 0.073 | 0.2118 | No |
| 36 | CSF1 |  |  | 2643 | 0.071 | 0.2137 | No |
| 37 | ECM1 |  |  | 2809 | 0.062 | 0.1988 | No |
| 38 | NCOA3 |  |  | 3270 | 0.038 | 0.1491 | No |
| 39 | CDC6 |  |  | 3392 | 0.032 | 0.1374 | No |
| 40 | APLP1 |  |  | 3430 | 0.030 | 0.1350 | No |
| 41 | KLF6 |  |  | 3447 | 0.029 | 0.1349 | No |
| 42 | CYFIP1 |  |  | 3515 | 0.026 | 0.1288 | No |
| 43 | HUWE1 |  |  | 3517 | 0.026 | 0.1303 | No |
| 44 | AHNAK |  |  | 3528 | 0.026 | 0.1306 | No |
| 45 | ENPP1 |  |  | 3541 | 0.025 | 0.1308 | No |
| 46 | SPRED2 |  |  | 3653 | 0.022 | 0.1195 | No |
| 47 | SLC39A8 |  |  | 3718 | 0.020 | 0.1134 | No |
| 48 | FURIN |  |  | 3719 | 0.019 | 0.1146 | No |
| 49 | TNFRSF1B |  |  | 3727 | 0.019 | 0.1149 | No |
| 50 | NRP1 |  |  | 3735 | 0.019 | 0.1152 | No |
| 51 | TIAM1 |  |  | 3782 | 0.016 | 0.1109 | No |
| 52 | SPRY4 |  |  | 3821 | 0.015 | 0.1075 | No |
| 53 | MAP6 |  |  | 3889 | 0.012 | 0.1007 | No |
| 54 | CCNE1 |  |  | 4201 | -0.001 | 0.0657 | No |
| 55 | UMPS |  |  | 4211 | -0.002 | 0.0648 | No |
| 56 | CCND2 |  |  | 4539 | -0.016 | 0.0289 | No |
| 57 | MYC |  |  | 4656 | -0.020 | 0.0170 | No |
| 58 | SERPINB6 |  |  | 4759 | -0.024 | 0.0069 | No |
| 59 | BMP2 |  |  | 4770 | -0.025 | 0.0072 | No |
| 60 | PDCD2L |  |  | 4983 | -0.032 | -0.0148 | No |
| 61 | PLSCR1 |  |  | 5043 | -0.034 | -0.0194 | No |
| 62 | GADD45B |  |  | 5106 | -0.037 | -0.0243 | No |
| 63 | CA2 |  |  | 5139 | -0.038 | -0.0257 | No |
| 64 | ST3GAL4 |  |  | 5342 | -0.046 | -0.0457 | No |
| 65 | UCK2 |  |  | 5343 | -0.046 | -0.0431 | No |
| 66 | PRAF2 |  |  | 5478 | -0.052 | -0.0552 | No |
| 67 | SNX9 |  |  | 5535 | -0.054 | -0.0583 | No |
| 68 | NDRG1 |  |  | 5645 | -0.060 | -0.0671 | No |
| 69 | EEF1AKMT1 |  |  | 5692 | -0.062 | -0.0687 | No |
| 70 | SOCS2 |  |  | 5753 | -0.064 | -0.0718 | No |
| 71 | SWAP70 |  |  | 5773 | -0.064 | -0.0702 | No |
| 72 | CDKN1C |  |  | 5823 | -0.066 | -0.0719 | No |
| 73 | FAH |  |  | 5845 | -0.067 | -0.0704 | No |
| 74 | GABARAPL1 |  |  | 5860 | -0.068 | -0.0680 | No |
| 75 | BHLHE40 |  |  | 5950 | -0.072 | -0.0739 | No |
| 76 | PLIN2 |  |  | 6129 | -0.081 | -0.0892 | No |
| 77 | RHOB |  |  | 6287 | -0.090 | -0.1017 | No |
| 78 | HIPK2 |  |  | 6320 | -0.091 | -0.1000 | No |
| 79 | DENND5A |  |  | 6342 | -0.092 | -0.0971 | No |
| 80 | MYO1C |  |  | 6459 | -0.099 | -0.1044 | No |
| 81 | RGS16 |  |  | 6543 | -0.104 | -0.1077 | No |
| 82 | AHR |  |  | 6678 | -0.112 | -0.1163 | No |
| 83 | ETV4 |  |  | 6761 | -0.118 | -0.1187 | No |
| 84 | CCND3 |  |  | 6881 | -0.128 | -0.1247 | No |
| 85 | CDC42SE2 |  |  | 6886 | -0.128 | -0.1177 | No |
| 86 | DCPS |  |  | 6900 | -0.129 | -0.1117 | No |
| 87 | ARL4A |  |  | 6930 | -0.131 | -0.1074 | No |
| 88 | CAPG |  |  | 6974 | -0.135 | -0.1044 | No |
| 89 | MAPKAPK2 |  |  | 6987 | -0.136 | -0.0979 | No |
| 90 | CASP3 |  |  | 7082 | -0.144 | -0.1002 | No |
| 91 | BATF3 |  |  | 7265 | -0.163 | -0.1112 | No |
| 92 | AHCY |  |  | 7274 | -0.164 | -0.1026 | No |
| 93 | BCL2L1 |  |  | 7308 | -0.168 | -0.0966 | No |
| 94 | RRAGD |  |  | 7321 | -0.170 | -0.0882 | No |
| 95 | RNH1 |  |  | 7414 | -0.183 | -0.0879 | No |
| 96 | ANXA4 |  |  | 7565 | -0.199 | -0.0933 | No |
| 97 | TNFRSF21 |  |  | 7627 | -0.208 | -0.0882 | No |
| 98 | GSTO1 |  |  | 8061 | -0.288 | -0.1202 | No |
| 99 | NCS1 |  |  | 8201 | -0.326 | -0.1170 | No |
| 100 | HOPX |  |  | 8257 | -0.341 | -0.1035 | No |
| 101 | ITGAE |  |  | 8507 | -0.438 | -0.1062 | No |
| 102 | GLIPR2 |  |  | 8547 | -0.457 | -0.0841 | No |
| 103 | PHLDA1 |  |  | 8805 | -0.655 | -0.0751 | No |
| 104 | ODC1 |  |  | 8839 | -0.697 | -0.0385 | No |
| 105 | GPX4 |  |  | 8966 | -0.946 | 0.0020 | No |
Table: GSEA details [plain text format]

  

Fig 2: HALLMARK\_IL2\_STAT5\_SIGNALING: Random ES distribution      
 Gene set null distribution of ES for **HALLMARK\_IL2\_STAT5\_SIGNALING**
